# Is the Integration of Additional Eccentric, Balance and Core Muscles Exercises into a Typical Soccer Program Effective in Improving Strength and Postural Stability?

**DOI:** 10.3390/sports9110147

**Published:** 2021-10-25

**Authors:** Konstantinos Dafkou, Chrysostomos Sahinis, Athanasios Ellinoudis, Eleftherios Kellis

**Affiliations:** Laboratory of Neuromechanics, Department of Physical Education and Sport Sciences at Serres, Aristotle University of Thessaloniki, 62110 Serres, Greece; sahinisc@phed-sr.auth.gr (C.S.); ellinoud@phed-sr.auth.gr (A.E.)

**Keywords:** eccentric training, hamstrings, balance, core stability

## Abstract

Soccer teams integrate specific exercises into their typical workout programs for injury prevention. The purpose of this study was to investigate whether the incorporation of a brief and supplementary training program that involves eccentric, balance, and core exercises into the weekly soccer schedule can cause positive neuromuscular adaptations. Twenty-one soccer players were randomly allocated to either a training (*n* = 11) or a control group (*n* = 10). All players followed their teams’ typical program, consisting of 4–5 soccer-specific sessions plus 1 match, weekly. Training group players additionally performed biweekly, hamstring eccentric, balance, and core stability exercises for 8 weeks. Isokinetic concentric and eccentric peak torque (PT) of the hamstrings and quadriceps, changes in the center of pressure (COP) during a 30 s single-leg stance, and a supine bridge (trunk stability) test were assessed before and after the intervention. After the intervention, a 27% increase in hamstring concentric PT and a 33% reduction in COP sway in the stance test, were observed for the training group only (*p* < 0.05). These improvements were significant only for the non-dominant leg. Furthermore, the control group displayed an increase in COP sway during the bridge test compared to baseline values (*p* < 0.05), which reflects a deterioration in postural balance over time. Consequently, incorporating small doses of hamstring eccentric, proprioception, and core stability exercises into a typical training program of youth soccer players improves strength and postural balance in the non-dominant leg, as well as core muscle performance.

## 1. Introduction

Hamstring tears, knee and, ankle ligament strains are common soccer injuries, with a high recurrence rate and rehabilitation time [1,2,3]. Eccentric exercises are routinely included in soccer training programs [4,5,6,7,8], as they can reduce hamstring injury rate [9]. Nevertheless, hamstring injuries remain high [2,10], and this may be related to the multifactorial etiology of such injury [1,11].

The capacity of trunk core muscles to effectively resist perturbations has been established as an injury risk factor [12], whereas additional balance training improves proprioception [13,14], which may also reduce injury risk [15]. Hence, core stability and postural balance can be part of an injury prevention program [12,16].

Previous studies [4,5,6,7,13,17,18] have reported improvements in individual fitness components (strength, flexibility, or balance) associated with injuries. Most interventions, however, have been applied independently from the regular soccer program [5,13,18], or their overall training dosage has been relatively high [4,5,17]. This might lead to low compliance with “additional” injury prevention exercises or part of them. The already increased training loads in elite soccer require time-efficient and multi-component injury prevention interventions. Hence, many teams incorporate such programs into their training schedule as a small part of their session.

To our knowledge, only two studies [6,7] have examined the effects of injury-prevention exercises that supplement regular soccer training, but they only used hamstring strengthening exercises [6,7]. Supplementary training programs that simultaneously develop several fitness elements linked with injury, but which can be easily incorporated into the typical program of a soccer team, are necessary. Therefore, our primary purpose was to investigate the effects of a multifaceted training program in addition to the soccer training on strength, balance, and core stability performance in young soccer players. An additional purpose of the study was to examine whether these exercises affect differently dominant (D) and non-dominant (ND) sides of athletes. We hypothesized that adding two extra training sessions per week would result in greater adaptations compared with the typical soccer training program alone.

## 2. Materials and Methods

### 2.1. Participants

Initially, 123 players from six U19 soccer teams were invited to participate in the study. Inclusion criteria were: (1) male players from the U19 semi-professional Championship, (2) age range 16–19 years, and (3) supervised training at least 3 times plus 1–2 games, weekly. Participants were excluded if they had a history of anterior cruciate ligament rupture, suffered from any lower limb musculoskeletal injury the previous year, were absent from training for 2 weeks for any reason, or were involved in any additional hamstring, core muscle, or balance training program in the preceding year.

Subsequently, 42 players did not meet the inclusion criteria, 12 refused to participate, and 48 were excluded because they met the exclusion criteria. Hence, 21 players participated after providing a signed written informed consent themselves, or having it provided by their parents or legal guardians (<18 years). These athletes were randomly assigned by a third blinded investigator, using a computer-generated program, to either a training group (TG) (*n* = 11, age: 17.7 ± 1.15 years; height: 179.2 ± 7.2 cm; mass 75.42 ± 8.4 kg; BMI: 23.24 ± 2.5) or a control group (CG) (*n* = 10, age: 18.1 ± 0.57 years; height: 177.8 ± 5.9 cm; mass 70.75 ± 9.5 kg; BMI: 22.75 ± 1.79).

### 2.2. Procedures

A familiarization session with all experimental procedures preceded the baseline measurements, during which we recorded the participants’ height, mass, age, D leg defined as the preferred leg to kick a ball, and playing position. Baseline measurements were completed 4–7 days before the start of the intervention program.

A 10-min warm-up on a stationary cycle ergometer (100 watts at 70 rpm) and dynamic stretching preceded the main measurement tests. Strength tests were performed using an isokinetic dynamometer (System 3, Biodex Medical Systems, Newark, CA, USA). The participants were seated on the chair with the hip joint flexed to 100° and the mechanical axis of the dynamometer aligned with the lateral femoral epicondyle. We set the knee range of motion from 90° of knee flexion to 0° (full extension) and completed a torque gravity correction procedure. The protocol included 3 submaximal and 3 maximal concentric and eccentric efforts at 30°/s, 180°/s, and 240°/s. Concentric strength assessment preceded the eccentric to avoid possible acute eccentric contraction effects. Both legs were evaluated in random order. There were 1- and 2-min resting intervals between sets. Of the 3 trials, the highest observed peak torque (PT) (Nm) was further analysed.

**Figure 1:**
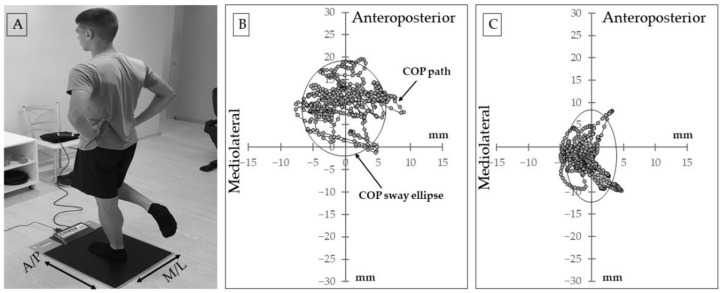
(**A**) Illustration of the single-leg stance test. (**B**) Typical example for a participant’s non-dominant leg center of pressure (COP) displacement during a 30-s trial, before the intervention. (**C**) Typical example for the same participant’s COP displacement during a 30-s trial after the intervention.

Single-leg balance was evaluated on a pressure platform (Comex SA, Loran Engineering Ltd., Bologna, Italy). Participants stood erect on one foot pointing forward to reference lines in the frontal and sagittal planes, as motionless as possible, with arms akimbo and the swinging leg at 90° knee flexion (Figure 1). Participants kept their sight on a fixed wall mark at approximately eye level. Two 30-s trials on each foot with a 2-min interval were performed. Pressure data were recorded at 50 Hz and the following varables were calculated using a computer program (FootChecker 4.0, Engineering S.r.I., Bologna, Italy): (1) the total COP path (TCOP) (mm), defined as the average distance of the COP from the reference lines, (2) average COP velocity (COPvel) (mm/s), calculated as the total length of the path of COP divided by the test trial time, (3) the standard deviation of COP (mm) in the anteroposterior (SDa) and mediolateral (SDm) axis, the COP sway area (mm^2^), and the COP sway ellipse (cm^2^), representing the smallest ellipse that will cover 95% of the points of the COP diagram [19].

**Figure 2:**
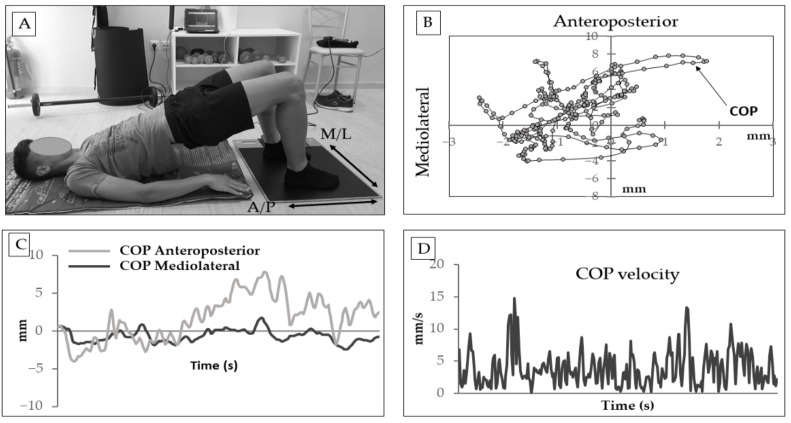
(**A**) Illustration of the bridge test which required the participants to place their feet on the pressure platform, while performing a bridge from the crook-lying position. (**B**) Typical example of center of pressure (COP) individual points during a 30-s trial, in the bridge test. (**C**) Typical example of anteroposterior and mediolateral COP displacement fluctuation, during a 30-s trial. (**D**) Typical example of COP velocity fluctuation during a 30-s trial.

To obtain an index of trunk movement stability, participants performed the bridge test [20,21] with their feet placed on the pressure platform (Figure 2). From crook-lying position, participants performed a bridge by lifting their pelvis to the point where shoulder, hip, and knee joints were aligned and remained as stable as possible. Participants were not allowed to use their arms to assist in pelvis stabilization and were instructed to rest them on the floor next to the body. If any assistance from the arms was noticed by the investigators, the trial was restarted. The measurement included two 30-s trials with a 2-min rest between them. Subsequently, COP path, sway, ellipse, and velocity were analyzed. The trial which showed the lowest COP displacement was further examined.

### 2.3. Intervention

The intervention was applied in the mid-season (competitive period) of the Championship. All (TG and CG) players followed their typical soccer team program, designed and supervised by the team coach, consisting of 4–5 training sessions, plus one game a week. Each session included warm-up activities (15–20 min), technical and tactical exercises (passing games, small-sided games, formation drills, full game scrimmage) (40–50 min), and cooling-down (10 min). A typical weekly schedule included an aerobic endurance-based session (interval shuttle-running (15″-15″), ball possession games), an explosive strength training session (plyometric hurdle-jumps combined with sprinting actions), and an anaerobic endurance training session (repeated sprint drills, high-intensity small-sided games). This program did not include lower extremity and trunk strengthening or proprioception exercises.

TG players (*n* = 11) completed 16 sessions of specific training, performed biweekly for 8 weeks, in addition to typical soccer training. The program included a hamstring eccentric exercise, five single-leg balance variations, and four core-muscle exercises. It was performed before the soccer-specific session under the supervision of a fitness coach.

**Figure 3:**
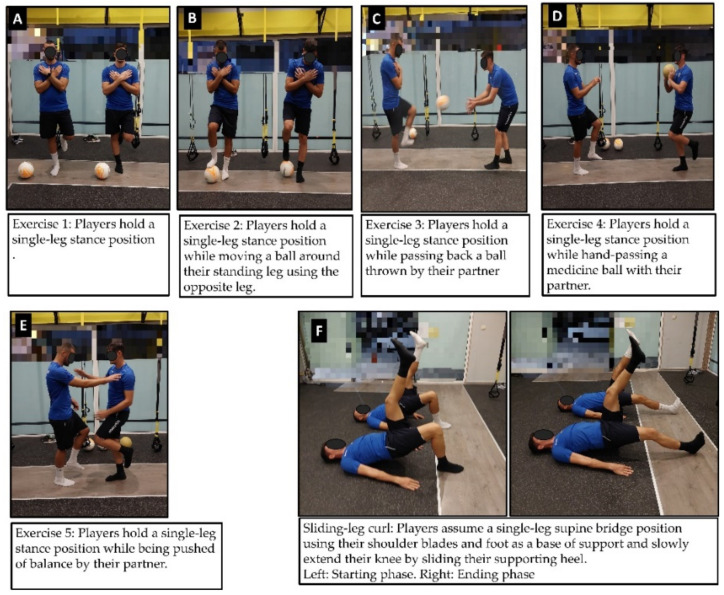
Illustration of balance (**A**–**E**) and sliding-leg curl (**F**) exercises used in the supplementary intervention program.

For eccentric strengthening participants performed the sliding-leg curl [22] (Figure 3). From a single-leg bridge position with the body supported on shoulder blades and one heel, wearing socks to reduce friction with the floor, the knee of the supporting leg was slowly extended (6–8 s to full extension) by sliding the foot forward, to eccentrically load the hamstrings. A repetition was completed after the knee was fully extended and the body was lowered to the floor. Players returned to the starting position by flexing the knee, while keeping the body on the floor to minimize concentric contraction. Initially, 2 sets of 6 reps on each leg were performed and gradually increased, to 4 sets of 10 reps on each leg, by week 6.

Balance exercises included several single-leg tasks on a stable surface and required the players to work in couples (Figure 3). Initially, the program consisted of 4 sets of 30-s attempts on each leg, to maintain single-leg stance, and gradually progressed to 6 sets of 45-s efforts and more complex tasks, including (a) kicking back a ball with their non-standing leg thrown by their partner, (b) moving a ball around their standing leg using the foot of the non-standing leg, (c) hand passing a medicine ball with their partner and, (d) being pushed off balance.

**Figure 4:**
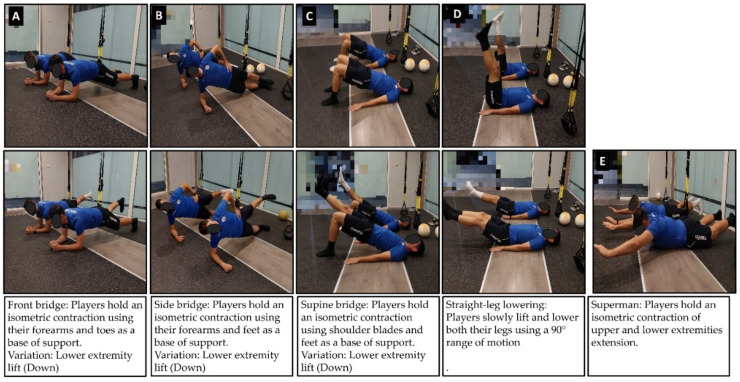
Illustration of core muscle exercises used in the intervention program.

Core stability exercises included front, side, and supine bridges, superman exercises [23], and straight-leg lowerings (Figure 4). At the beginning of the intervention, the players performed 3 sets of 30-s efforts for static exercises and 10 repetitions of straight-leg lowerings. Progression was built every 2 weeks by adding (a) an extra set, (b) 15-s extra time and 5 extra reps, and (c) unilateral variations of each exercise, where applicable.

### 2.4. Statistical Analysis

Using SPSS (version 25, IBM) and the Kolmogorov–Smirnov test, all data were found to be normally distributed. Two-way split-plot analysis of variance (ANOVA) were applied to examine the interaction effects of time (pre-post) and group (TG-CG) on PT in each angular velocity test as well as in each COP variable, on each leg. Alpha level was set a priori at 0.05 and when a significant interaction effect was detected, we used post-hoc Tukey tests to determine which comparisons differed. Partial eta squared effect sizes (η^2^) for interaction effects were classified as small 0.01–0.06; medium 0.06–0.14; and large ≥0.14 [24]. Sample size was determined by a priori power analysis (G*Power version 3.1.9.4; Heinrich-Heine-Universität Düsseldorf, Düsseldorf, Germany) using data from previous studies [18,25] examining similar strengthening interventions. To achieve 80% statistical power with an alpha level of less than 0.05 revealed a minimum of 16 participants. To allow for potential dropout, we aimed to recruit a minimum of 20 participants.

## 3. Results

### 3.1. Hamstring Strength

The average group values for hamstring concentric and eccentric condition are presented in Table 1. The ANOVA showed a significant time by group interaction effect on concentric PT at 30°/s in the ND leg, with large effect sizes (F_1,18_ = 5.84, *p* = 0.02, η^2^ = 0.24), but no significant effects in the D leg (*p* > 0.05) (Table 1). Post hoc analysis showed that PT of the ND leg significantly increased after the intervention in TG only (*p* < 0.05). No significant interaction or main effects were observed in the eccentric PT variables (*p* > 0.05).

### 3.2. Quadriceps Strength

Table 2 presents the mean values for quadriceps concentric and eccentric PT for each testing condition. There was a significant time by group interaction effect on concentric PT at 240°/s in the D leg (F_1,18_ = 6.16, *p* = 0.02, η^2^ = 0.25) (Table 2). Post hoc analysis showed that PT in the D leg at 240°/s significantly increased after the intervention in the TG only (Table 2, *p* < 0.05). Similarly, there was a statistically significant interaction effect on the eccentric PT at 30°/s in the ND leg (F_1,18_ = 4.69, *p* = 0.04, η^2^ = 0.20). Post-hoc analysis indicated that the CG showed a decrease in ND leg eccentric PT at 30°/s after the intervention (*p* < 0.05).

### 3.3. Single-Leg Balance

**Figure 5:**
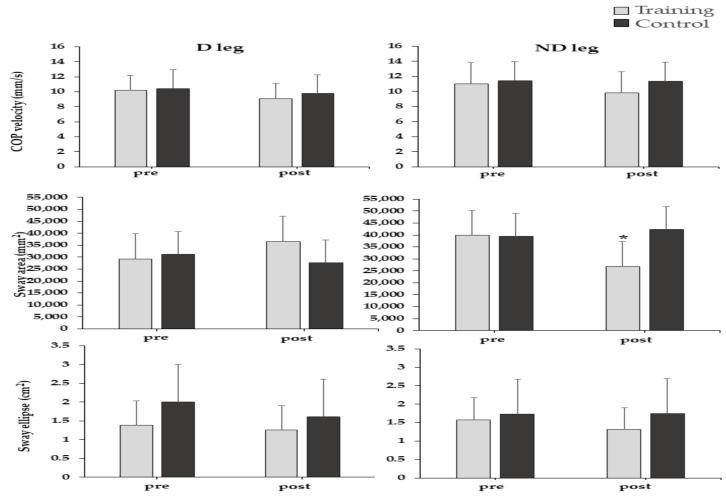
Mean values of the center of pressure (COP) variables during the single-leg stance test. (error bars indicate standard deviation). * Significantly (*p* < 0.05) different with pre-training.

Average group values for COP velocity, sway and ellipse are presented in Figure 5. There was a significant time by group interaction effect on COP sway area of the ND leg, (F_1,18_ = 8.63, *p* = 0.009, η^2^ = 0.32), but not for the D leg (*p* > 0.05) (Figure 5). Post hoc analysis showed that COP sway area of the ND leg significantly decreased after the intervention only in the TG (*p* < 0.05). Furthermore, there was a significant main effect of time on COP mediolateral sway of the ND leg (F_1,18_ = 6.84, *p* = 0.017, η^2^ = 0.27), but not for the D leg (*p* > 0.05).

Group values for COP displacement and SD during single leg test are presented in Table 3. The ANOVA showed non-significant interaction or main effects for any of these variables (*p* > 0.05) (Table 3).

### 3.4. Bridge Test

**Figure 6:**
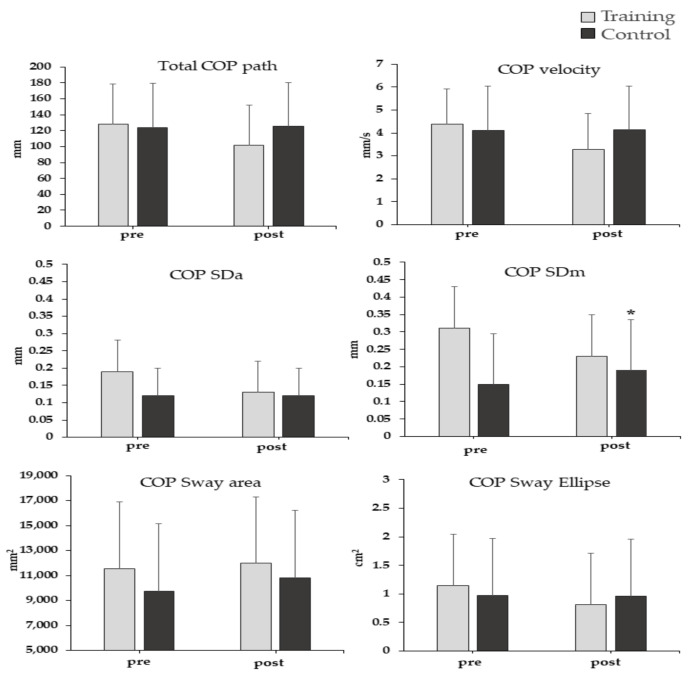
Mean values of the center of pressure (COP) variables during the bridge test. (Error bars indicate standard deviation). * Significantly (*p* < 0.05) different with pre-training.

The average group values for each COP variable are illustrated in Figure 6. There was a significant time by group interaction effect on COP mediolateral sway (COP SDm), (F_1,18_ = 9.87, *p* = 0.006, η^2^ = 0.35) (Figure 6). Post hoc analysis showed that COP sway on the mediolateral axis significantly increased in the CG, compared to baseline values (*p* < 0.05). No significant interaction or main effects were observed in the other COP variables (*p* > 0.05).

## 4. Discussion

The main findings of this study were that players who followed a supplementary program that consisted of core, eccentric and postural balance exercises showed improvements in hamstring strength and single-leg balance on the ND leg. Furthermore, the TG showed no decrement in instability during the bridge test during the intervention period as opposed to a lower performance which was seen in the CG. These findings partially confirm our hypotheses.

A 27% increase was observed in concentric hamstring ND strength in TG (Table 1). Since there are concerns that bilateral exercises like Nordic might provide a lower stimulus for the weaker leg leading to asymmetries [26], we implemented a unilateral hamstring exercise which can be performed without any equipment or assistance and induces high hamstring activation [22]. The observation of greater gains to the ND leg is in line with previous studies [25,27] who also reported increased PT in the ND leg after eccentric hamstring training. In particular, Delextrat et al. [25] observed increments in eccentric PT only for the ND leg after Nordic or eccentric leg curl training, while Brito et al. [27] reported improvements in both concentric and eccentric PT in the ND leg after 10 weeks of FIFA 11+. This non-uniform adaptation to training may be due to two main reasons: ND leg may be less adapted to the eccentric exercise than the D leg by reflecting changes to greater intensity, or on the other hand, the pattern of favorable gains in the ND leg could be related to a potential between-limb asymmetry in PT, even though in our study, baseline PT values of D and ND leg were not statistically different. However, in our study, strength improvements were not mode specific, as the eccentric training significantly increased concentric strength only.

Contrary to previous studies that found increments (11–21%) in hamstring eccentric strength following high volume eccentric exercise programs for 4 [4,18] or 10 [5] weeks, the TG indicated a small (ranging from 4.8 to 10.9%) but insignificant improvement in eccentric strength (Table 1). This is probably because these studies implemented programs with a greater training dosage (intensity, exercises, repetitions) compared to our program. Our results also contradict those of previous studies implementing supplementary training programs in soccer players [6,7]. Mendiguchia et al. [6] found a 15% eccentric torque increase at 60°/s following a supplementary hamstring strengthening program biweekly, for 7 weeks. Similarly, Askling et al. [7], observed eccentric strength increases of 19% after 16 sessions of additional eccentric overload training on a Yo-Yo flywheel ergometer during the preseason. While strength improvements are largely mode-specific, the magnitude of adaptations probably depends on the exercise loading and participants’ training level [28]. These factors may explain the absence of eccentric strength adaptations in our study as opposed to significant increases observed by others. First, previous studies implemented supplementary hamstring strengthening programs which consisted of a greater training dosage (more exercises, greater intensity, and repetitions) than the bodyweight sliding-leg curl exercise applied in this study. Additional eccentric overload above the concentric maximum is crucial for substantial eccentric strength gains, especially in trained athletes [28,29]. Consequently, training with sliding-leg curl for 8 weeks, biweekly is probably not effective enough to provide the eccentric overload required for eccentric strength development, in well-trained players. Second, the participants’ pre-intervention eccentric strength values (Table 1) were about 1.5 to 3 times greater than those reported by previous studies [4,7,25]. With such high levels of eccentric strength at baseline, the absence of eccentric strength adaptations is not a surprise. Finally, both groups followed regular soccer training which included aerobic, explosive and plyometric as well as sprinting and endurance exercises. This influenced training adaptations of both groups and, possibly, alleviated or integrated the adaptations due to the additional exercises performed by the TG.

Another interesting finding of this study was that the TG displayed an increase in concentric quadriceps D strength at 240°/s (Table 2), while the CG displayed a decrement in eccentric quadriceps ND strength at 30°/s (Table 2). A previous study [6] also reported 4.4% and 8.1% increases in quadriceps concentric strength, at D and ND legs, respectively, after 7 weeks of additional hamstring-emphasized training. However, they implemented a variety of eccentric exercises (lunges, hip thrust, deadlift) as well as plyometrics and sprints into their intervention, which could explain the improvements in quadriceps strength. Conversely, Clark et al. [30], observed an 11.3% reduction in concentric quadriceps strength, after 4 weeks of Nordic training, translating their findings into possible alterations in the viscoelastic properties (increased stiffness) and antagonistic activation of the hamstring muscle. Indeed, these neuromuscular adaptations of the hamstrings following the eccentric training could have affected the force output of quadriceps muscles. Our study was the first that assessed both concentric and eccentric quadriceps strength, at different angular velocities, following hamstring eccentric training, so the observation of a reduction in eccentric quadriceps strength after 8 weeks of soccer training only, is a unique finding.

The TG displayed a decrease in ND leg COP sway area during the single-leg test (Figure 5), which indicates an improvement in static balance. This is in agreement with previous research studies that applied injury prevention programs, such as FIFA 11+ [17], HarmoKnee [17], or proprioception training [31] and reported improvements in both static [13,17,31] and dynamic [17,31] balance tasks. In contrast, players, who do not perform balance exercises do not show balance improvements [13,17,31]. Despite our protocol being less intense than in previous studies [13,17,31], our results indicate that performing additional exercises biweekly, as part of supplementary training, is beneficial for static balance of soccer players.

Interestingly, significant improvement in balance was noted for the ND leg only (Figure 5), even though soccer players usually display greater balance performance in the ND leg [32]. Despite that one would expect better adaptations of balance training in the “less task-trained” leg, i.e., the D leg, the exact opposite was observed. A previous study with soccer players [13] also reported a trend for greater balance improvement in the ND leg, after training. It is possible that the ND leg becomes more adaptable to balance training after it is functionally challenged in single-leg tasks during training [13]. Additionally, our findings are in line with those of Sebastia-Amat [14], who also observed greater improvements in monopedal stance after balance training in the ND leg of volley-ball players. Previous authors [14,33] have attributed these favorable gains in the ND leg to “muscular reinforcement of the weakest limb as a consequence of the training program”. Indeed, the fact that our participants displayed improvements in both strength and stability of the ND leg could support this theory.

Players that did not follow the supplementary exercise program (CG) showed an increase in COP mediolateral sway during the bridge test (Figure 6). This means that the body during the bridge test became less stable for the group that did not perform additional core exercises (Figure 6). While typical soccer training included exercises that enhance trunk stabilization, there were no specific core stability exercises like those performed by the TG. Inadequately trained core muscles may fail to resist trunk rotational forces during bridge test and present greater pelvic tilt in the transverse plane [20]. There is evidence that a greater pelvic instability may cause perturbations in CG and lead to changes in COP amplitude in the mediolateral axis [34]. Hence, integration of core exercises into a soccer training program may serve to maintain trunk stability of the players during a typical competitive season. There is also a possibility that bridge test scores have been influenced by hamstring training exercises in the experimental group. However, studies have shown that during the bridge test, hamstring, gluteus maximus and the rectus abdominis activation is very small [35]. Furthermore, since participants in the experimental group performed task-specific (core muscle) exercises, such as bridges, it is highly unlikely that hamstring eccentric training was superior enough to influence performance during the bridge test.

The study limitations include the lack of follow-up measurements to determine how long the observed training adaptations would last. Second, while the sliding-leg exercise did not require any equipment, quantification of the effort to resist sliding was not possible. Third, the study sample was small, and participants were male U19 soccer players, which limits the generalization of the findings to other populations. The implementation of the bridge test can provide an indirect estimation of the core muscles’ function. Assessing core muscles’ contracting properties or activation using electromyography or ultrasound would be a more representative measure. Future studies should incorporate more core-specific testing procedures.

There are some implications of the present study. It is obvious that a greater training dosage of the supplementary and regular soccer training program results in greater training adaptations. However, typical soccer programs involve many intense weekly sessions to meet the high physiological demands of modern soccer. For this reason, soccer players are often hesitant to perform supplementary exercises, especially when they compete at a lower level (amateur or semi-professionals). Hence, compliance with injury prevention guidelines is a common problem in soccer [10]. Therefore, we implemented an exercise program comprising various fitness elements (strength, postural balance, trunk stability) that would be more practically convenient and less time consuming. Training dosage was set to an additional number of two sessions a week and a reasonable number of exercises. Our results showed some positive adaptations in strength, postural balance, and trunk stability following a supplementary exercise program. Some of the improvements could have been due to the learning effect. However, if there was no impact by the supplementary training on isokinetic parameters, balance and core function, both groups should have shown similar adaptations (and, hence, learning effects). It is unclear though, whether these adaptations are adequate for injury prevention. Future research on attempting simple and easily adapted supplementary programs and monitory injury characteristics of those players is guaranteed.

## 5. Conclusions

Young soccer players, who performed the eccentric sliding-leg curl exercise, unipedal balance exercises, and core stability exercises along with their typical soccer training showed improvements in concentric strength and balance on their ND leg after 8 weeks, while they maintained core stability performance as opposed to the CG. Integrating small “doses” of eccentric, proprioception, and core training into a typical soccer exercise program at elite youth level may improve fitness parameters that are often linked with injury occurrence. Such programs can be easily integrated into a typical soccer program during the competitive season.

## Figures and Tables

**Table 1:** Mean ± SD hamstring concentric and eccentric peak torque (Nm) of the training and control group, before and after the intervention.

	Concentric Peak Torque			Eccentric Peak Torque		
	Before	After		Before	After	
Group	x ± SD	x ± SD		x ± SD	x ± SD	
	240°/s, Dominant Leg		% change	240°/s, Dominant Leg		% change
Training	90.77 ± 15.03	95.39 ± 16.14	5.09 ± 7.38	185.22 ± 35.88	197.02 ± 25.05	6.37 ± 30.18
Control	88.42 ± 15.27	87.04 ± 20.61	−1.56 ± 34.97	187.24 ± 30.01	186.41 ± 30.55	−0.42 ± 1.79
240°/s, Non-dominant Leg				240°/s, Non-dominant Leg		
Training	87.14 ± 21.57	88.49 ± 13.76	1.55 ± 36.20	173.51 ± 39.57	187.04 ± 19.39	7.80 ± 50.99
Control	84.12 ± 11.04	86.77 ± 13.97	3.15 ± 26.53	171.60 ± 24.51	179.77 ± 40.70	4.76 ± 63.19
180°/s, Dominant Leg				180°/s, Dominant Leg		
Training	101.37 ± 19.67	103.39 ± 18.45	1.99 ± 6.20	180.73 ± 31.80	189.45 ± 40.01	4.82 ± 25.81
Control	107.21 ± 25.32	101.3 ± 16.79	−5.51 ± 33.68	186.94 ± 39.43	183.28 ± 41.20	−1.96 ± 4.48
180°/s, Non-dominant Leg				180°/s, Non-dominant Leg		
Training	96.61 ± 19.43	101.72 ± 18.47	5.29 ± 4.94	176.51 ± 38.46	195.81 ± 31.81	10.93 ± 17.29
Control	113.63 ± 27.28	109.85 ± 30.44	−3.33 ± 11.58	174.94 ± 28.37	178.75 ± 34.2	2.18 ± 20.54
30°/s, Dominant Leg				30°/s, Dominant Leg		
Training	153.80 ± 51.86	149.62 ± 43.95	−2.72 ± 14.95	199.88 ± 45.40	210.15 ± 39.50	5.14 ± 12.99
Control	157.78 ± 32.99	144.56 ± 36.37	−8.38 ± 10.24	214.65 ± 39.61	211.66 ± 48.81	−1.39 ± 23.22
30°/s, Non-dominant Leg				30°/s, Non-dominant Leg		
Training	139.55 ± 34.24	177.57 ± 58.56 *	27.24 ± 71.02	188.31 ± 39.96	198.90 ± 39.01	5.62 ± 2.37
Control	148.3 ± 37.15	127.94 ± 50.97	−13.33 ± 37.20	188.98 ± 29.13	181.4 5± 47.30	−3.98 ± 62.37

* indicates a significant difference compared to pre-training value; significance level: *p* < 0.05.

**Table 2:** Mean ± SD quadriceps concentric and eccentric peak torque (Nm) of the training and control group, before and after the intervention.

	Concentric Peak Torque			Eccentric Peak Torque		
	Before	After		Before	After	
Group	x ± SD	x ± SD		x ± SD	x ± SD	
	240°/s, Dominant Leg		% change	240°/s, Dominant Leg		% change
Training	128.89 ± 31.66	146.07 ± 22.63 *	13.32 ± 28.52	328.74 ± 59.31	292.64 ± 115.41	10.97 ± 93.89
Control	137.99 ± 21.31	132.48 ± 14.35	−3.99 ± 32.66	306.16 ± 70.35	295.41 ± 81.67	3.59 ± 16.15
240°/s, Non-dominant Leg				240°/s, Non-dominant Leg		
Training	135.81 ± 24.14	146.46 ± 21.55	7.84 ± 10.72	309.70 ± 81.58	320.43 ± 77.51	3.55 ± 4.93
Control	129.4 ± 20.20	127.01 ± 23.29	−1.85 ± 15.29	312.21 ± 66.19	298.06 ± 69.73	4.48 ± 4.54
180°/s, Dominant Leg				180°/s, Dominant Leg		
Training	160.16 ± 28.98	174.33 ± 27.17	8.84 ± 6.24	312.56 ± 69.22	298.04 ± 89.38	4.64 ± 28.98
Control	163.19 ± 22.16	160.68 ± 18.99	−1.53 ± 14.3	289.92 ± 80.84	286.77 ± 93.50	1.03 ± 16.25
180°/s, Non-dominant Leg				180°/s, Non-dominant Leg		
Training	162.01 ± 18.35	169.38 ± 24.06	4.54 ± 31.11	309.85 ± 67.64	290.88 ± 69.35	6.14 ± 2.98
Control	156.45 ± 20.87	156.07 ± 21.93	−0.24 ± 5.08	303.53 ± 67.44	290.49 ± 77.56	4.29 ± 14.92
30°/s, Dominant Leg				30°/s, Dominant Leg		
Training	276.77 ± 54.06	273.20 ± 42.68	−1.28 ± 21.05	369.86 ± 47.55	373.4 ± 33.12	1.08 ± 29.78
Control	263.71 ± 39.35	253.55 ± 50.20	−3.85 ± 25.65	333.91 ± 54.27	329.02 ± 83.33	1.20 ± 53.53
30°/s, Non-dominant Leg				30°/s, Non-dominant Leg		
Training	277.52 ± 46.11	277.94 ± 38.37	0.15 ± 16.78	365.15 ± 41.71	372.62±50.48	1.91 ± 21.95
Control	256.57 ± 33.45	244.89 ± 43.57	−4.55 ± 30.25	352.96 ± 60.59	334.98 ± 62.10 *	5.11 ± 3.33

* indicates a significant difference compared to pre-training value; significance level: *p* < 0.05.

**Table 3:** Mean± SD of COP variables during the single-leg test for the training and control group, before and after the intervention.

	Before	After		Group × Time Interaction
Group	x ± SD	x ± SD		
	TCOP (mm), Dominant Leg		% change	
Training	305.61 ± 89.80	272.91 ± 60.97	−10.7 ± 32.10	NS
Control	313.04 ± 95.26	287.9 ± 66.73	−8.03 ± 29.94	
TCOP (mm), Non-dominant Leg				
Training	330.09 ± 71.01	292.56 ± 102.64	−11.6 ± 44.54	NS
Control	342.74 ± 74.18	339.55 ± 91.77	−1.07 ± 23.71	
SDa (mm), Dominant Leg				
Training	0.41 ± 0.10	0.34 ± 0.11	−17.07 ± 11.13	NS
Control	0.36 ± 0.08	0.34 ± 0.05	−5.55 ± 37.5	
SDa (mm), Non-dominant Leg				
Training	0.39 ± 0.10	0.35 ± 0.14	−10.25 ± 10.40	NS
Control	0.40 ± 0.08	0.39 ± 0.09	−2.50 ± 12.50	
SDm (mm), Dominant Leg				
Training	0.29 ± 0.11	0.23 ± 0.09	−20.68 ± 18.18	NS
Control	0.36 ± 0.38	0.22 ± 0.06	−38.88 ± 54.21	
SDm (mm), Non-dominant Leg				
Training	0.30 ± 0.07	0.23 ± 0.07	−23.33 ± 10.31	NS
Control	0.31 ± 0.09	0.25 ± 0.07	−19.35 ± 22.22	

Abbreviations: NS = non-significant, S = significant.

## Data Availability

Data are provided upon request.
